# (*E*)-2-Methyl-3-phenyl­selanyl-4-(phenyl­sulfin­yl)oct-3-en-2-ol

**DOI:** 10.1107/S1600536813032182

**Published:** 2013-12-04

**Authors:** Jinglan Wu, Chen Chen

**Affiliations:** a College of Biotechnology and Pharmaceutical Engineering, Nanjing University of Technology, Nanjing 210009, People’s Republic of China; b National Engineering Technique Research Center for Biotechnology, Nanjing 211816, People’s Republic of China; c Research Institute of Benzene Chemical, Research Institute of Nanjing Chemical Industry Group, No. 699 Geguan Road, Dachang, Nanjing 210044, People’s Republic of China

## Abstract

In the title compound, C_21_H_26_O_2_SSe, the S atom adopts a pyramidal geometry (bond-angle sum = 304°) and the *n*-butyl chain shows an extended conformation. An intra­molecular C—H⋯O hydrogen bond closes an *S*(8) ring. In the crystal, inversion dimers are formed with molecules linked by pairs of O—H⋯O=S hydrogen bonds, generating *R*_2_^2^(14) loops. Weak C—H⋯O inter­actions also occur.

## Related literature   

For background to the title compound, see: Uma *et al.* (2003 ▶). For the synthesis, see: He *et al.* (2007 ▶).
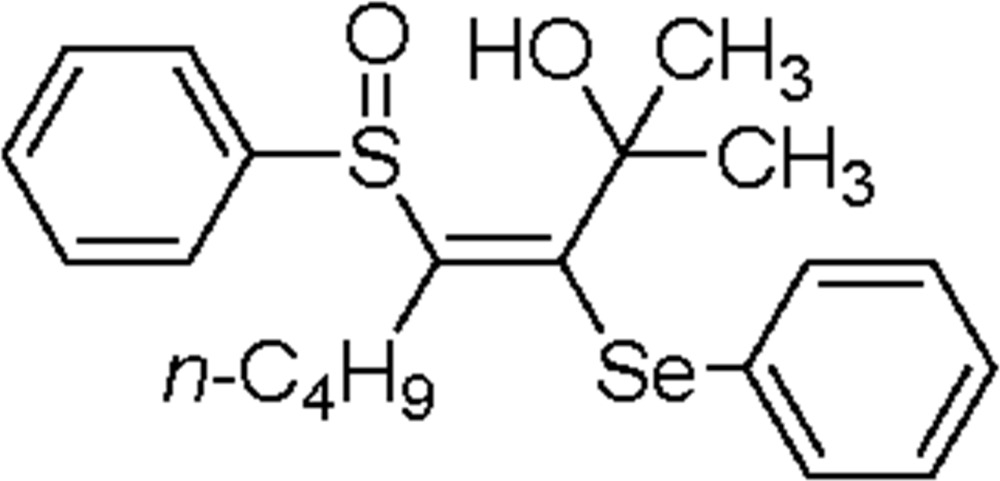


## Experimental   

### 

#### Crystal data  

C_21_H_26_O_2_SSe
*M* *_r_* = 421.44
Monoclinic, 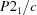

*a* = 12.869 (3) Å
*b* = 19.445 (4) Å
*c* = 8.3702 (18) Å
β = 100.280 (4)°
*V* = 2061.0 (8) Å^3^
*Z* = 4
Mo *K*α radiation
μ = 1.93 mm^−1^
*T* = 293 K
0.49 × 0.20 × 0.05 mm

#### Data collection  

Bruker SMART CCD diffractometer
Absorption correction: multi-scan (*SADABS*; Bruker, 2001 ▶) *T*_min_ = 0.590, *T*_max_ = 1.000
11048 measured reflections
4029 independent reflections
2554 reflections with *I* > 2σ(*I*)
*R*_int_ = 0.130

#### Refinement  

*R*[*F* ^2^ > 2σ(*F* ^2^)] = 0.086
*wR*(*F* ^2^) = 0.230
*S* = 1.01
4029 reflections
233 parameters
1 restraint
H atoms treated by a mixture of independent and constrained refinement
Δρ_max_ = 1.99 e Å^−3^
Δρ_min_ = −0.56 e Å^−3^

### 

Data collection: *SMART* (Bruker, 2001 ▶); cell refinement: *SAINT* (Bruker, 2001 ▶); data reduction: *SAINT*; program(s) used to solve structure: *SHELXS97* (Sheldrick, 2008 ▶); program(s) used to refine structure: *SHELXL97* (Sheldrick, 2008 ▶); molecular graphics: *SHELXTL* (Sheldrick, 2008 ▶); software used to prepare material for publication: *SHELXTL*.

## Supplementary Material

Crystal structure: contains datablock(s) I, cd26300. DOI: 10.1107/S1600536813032182/hb7167sup1.cif

Structure factors: contains datablock(s) I. DOI: 10.1107/S1600536813032182/hb7167Isup2.hkl

Supporting information file. DOI: 10.1107/S1600536813032182/hb7167Isup3.cml

Additional supporting information:  crystallographic information; 3D view; checkCIF report

## Figures and Tables

**Table 1:** Hydrogen-bond geometry (Å, °)

*D*—H⋯*A*	*D*—H	H⋯*A*	*D*⋯*A*	*D*—H⋯*A*
O1—H1⋯O2^i^	0.85 (9)	1.91 (8)	2.737 (6)	167 (10)
C11—H11⋯O1	0.93	2.50	3.180 (7)	130
C15—H15⋯O2^ii^	0.93	2.60	3.463 (7)	155

Symmetry codes: (i) 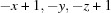; (ii) 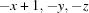.

**Fig. 1.**
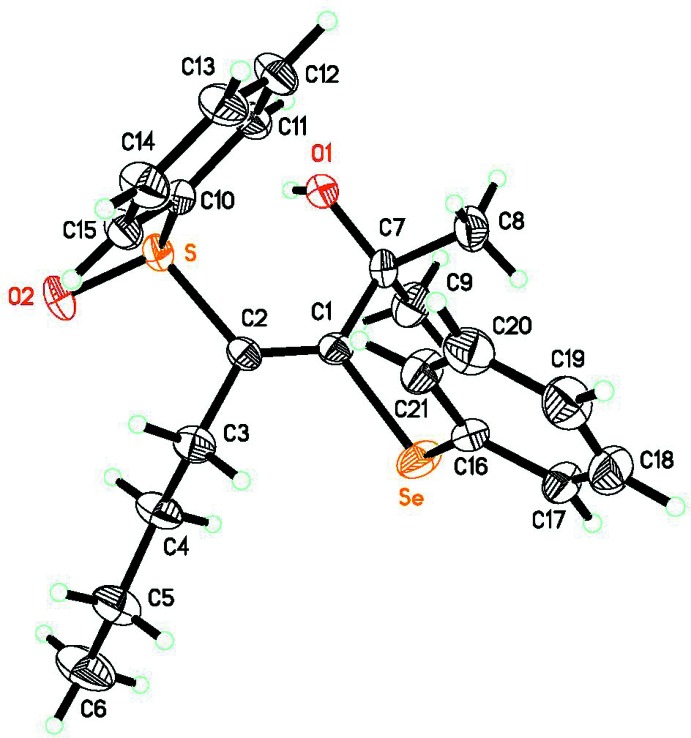
The molecular structure of (I), with displacement ellipsoids drawn at the 50% probability level.

**Fig. 2.**
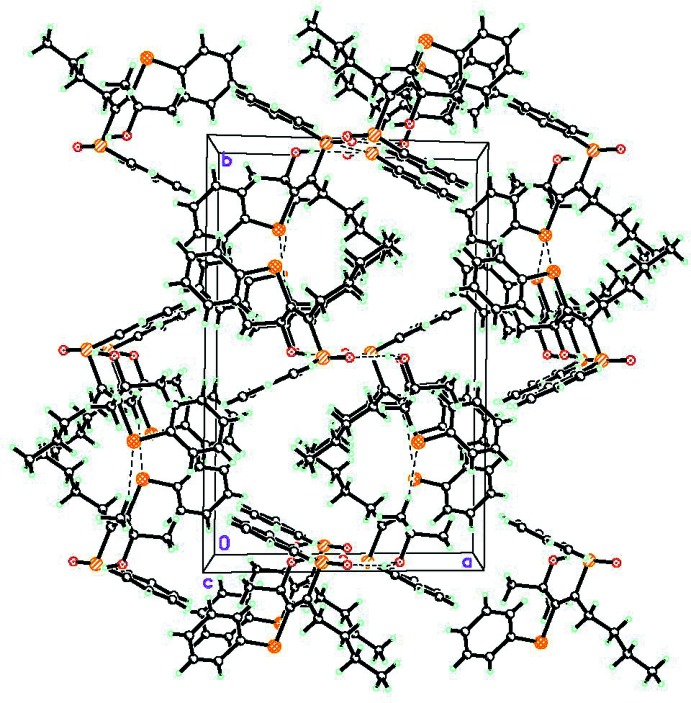
A packing diagram of (I). O—H···O and C—H···O hydrogen bonds are shown as dashed lines.

## supplementary crystallographic information

### 1. Experimental

The title compund was synthesized according to the published procedure (He *et al.*, 2007). Colourless blocks were obtained by dissolving it (0.5 g) in tetrahydrofuran (20 ml) and evaporating the solvent slowly at room temperature for about 10 d.

### 2. Refinement

H atoms were positioned geometrically and refined as riding groups, with C—H = 0.93 Å for aromatic H, and constrained to ride on their parent atoms, with *U*_iso_(H) = *xU*_eq_(C), where *x* = 1.2 for aromatic H, and *x* = 1.5 for other H.

### Crystal data

**Table:**

C_21_H_26_O_2_SSe	*F*(000) = 872
*M**_r_* = 421.44	*D*_x_ = 1.358 Mg m^−^^3^
Monoclinic, *P*2_1_/*c*	Mo *K*α radiation, λ = 0.71073 Å
Hall symbol: -P 2ybc	Cell parameters from 2566 reflections
*a* = 12.869 (3) Å	θ = 5.3–46.3°
*b* = 19.445 (4) Å	µ = 1.93 mm^−^^1^
*c* = 8.3702 (18) Å	*T* = 293 K
β = 100.280 (4)°	Block, colorless
*V* = 2061.0 (8) Å^3^	0.49 × 0.20 × 0.05 mm
*Z* = 4	

### Data collection

**Table:**

Bruker SMART CCD diffractometer	4029 independent reflections
Radiation source: fine-focus sealed tube	2554 reflections with *I* > 2σ(*I*)
Graphite monochromator	*R*_int_ = 0.130
ω scans	θ_max_ = 26.0°, θ_min_ = 1.6°
Absorption correction: multi-scan (*SADABS*; Bruker, 2001)	*h* = −15→15
*T*_min_ = 0.590, *T*_max_ = 1.000	*k* = −23→23
11048 measured reflections	*l* = −7→10

### Refinement

**Table:**

Refinement on *F*^2^	Primary atom site location: structure-invariant direct methods
Least-squares matrix: full	Secondary atom site location: difference Fourier map
*R*[*F*^2^ > 2σ(*F*^2^)] = 0.086	Hydrogen site location: inferred from neighbouring sites
*wR*(*F*^2^) = 0.230	H atoms treated by a mixture of independent and constrained refinement
*S* = 1.01	*w* = 1/[σ^2^(*F*_o_^2^) + (0.1331*P*)^2^] where *P* = (*F*_o_^2^ + 2*F*_c_^2^)/3
4029 reflections	(Δ/σ)_max_ < 0.001
233 parameters	Δρ_max_ = 1.99 e Å^−^^3^
1 restraint	Δρ_min_ = −0.56 e Å^−^^3^

### Special details

**Table:**

Geometry. All e.s.d.'s (except the e.s.d. in the dihedral angle between two l.s. planes) are estimated using the full covariance matrix. The cell e.s.d.'s are taken into account individually in the estimation of e.s.d.'s in distances, angles and torsion angles; correlations between e.s.d.'s in cell parameters are only used when they are defined by crystal symmetry. An approximate (isotropic) treatment of cell e.s.d.'s is used for estimating e.s.d.'s involving l.s. planes.
Refinement. Refinement of *F*^2^ against ALL reflections. The weighted *R*-factor *wR* and goodness of fit *S* are based on *F*^2^, conventional *R*-factors *R* are based on *F*, with *F* set to zero for negative *F*^2^. The threshold expression of *F*^2^ > σ(*F*^2^) is used only for calculating *R*-factors(gt) *etc*. and is not relevant to the choice of reflections for refinement. *R*-factors based on *F*^2^ are statistically about twice as large as those based on *F*, and *R*- factors based on ALL data will be even larger.

### Fractional atomic coordinates and isotropic or equivalent isotropic displacement parameters (Å^2^)

**Table:**

	*x*	*y*	*z*	*U*_iso_*/*U*_eq_	
Se	0.75777 (6)	0.20048 (3)	0.48905 (9)	0.0587 (3)	
S	0.58519 (11)	−0.00576 (8)	0.36854 (17)	0.0387 (4)	
O1	0.7008 (3)	0.0066 (2)	0.6746 (5)	0.0426 (10)	
O2	0.4924 (3)	−0.0048 (2)	0.2318 (5)	0.0492 (11)	
C1	0.7020 (4)	0.1084 (3)	0.5070 (7)	0.0386 (13)	
C2	0.6377 (4)	0.0827 (3)	0.3772 (7)	0.0373 (13)	
C3	0.5957 (5)	0.1213 (3)	0.2208 (7)	0.0491 (15)	
H3A	0.5782	0.0886	0.1325	0.059*	
H3B	0.6504	0.1516	0.1951	0.059*	
C4	0.4977 (6)	0.1639 (4)	0.2346 (8)	0.0600 (18)	
H4A	0.4414	0.1332	0.2520	0.072*	
H4B	0.5138	0.1940	0.3282	0.072*	
C5	0.4604 (6)	0.2067 (4)	0.0850 (10)	0.071 (2)	
H5A	0.4431	0.1764	−0.0080	0.086*	
H5B	0.5177	0.2364	0.0662	0.086*	
C6	0.3650 (8)	0.2508 (5)	0.0966 (13)	0.101 (3)	
H6A	0.3091	0.2222	0.1211	0.151*	
H6B	0.3419	0.2738	−0.0049	0.151*	
H6C	0.3836	0.2843	0.1812	0.151*	
C7	0.7396 (4)	0.0751 (3)	0.6735 (7)	0.0424 (14)	
C8	0.8601 (5)	0.0684 (4)	0.7106 (9)	0.0636 (19)	
H8A	0.8812	0.0461	0.8136	0.095*	
H8B	0.8914	0.1134	0.7144	0.095*	
H8C	0.8833	0.0417	0.6272	0.095*	
C9	0.7015 (6)	0.1181 (4)	0.8048 (8)	0.0637 (19)	
H9A	0.6257	0.1193	0.7848	0.096*	
H9B	0.7283	0.1641	0.8027	0.096*	
H9C	0.7265	0.0980	0.9094	0.096*	
C10	0.6901 (4)	−0.0421 (3)	0.2807 (6)	0.0362 (12)	
C11	0.7778 (5)	−0.0700 (3)	0.3809 (7)	0.0449 (14)	
H11	0.7810	−0.0713	0.4928	0.054*	
C12	0.8597 (5)	−0.0957 (4)	0.3142 (8)	0.0545 (17)	
H12	0.9184	−0.1142	0.3813	0.065*	
C13	0.8556 (5)	−0.0943 (3)	0.1477 (8)	0.0534 (17)	
H13	0.9117	−0.1112	0.1032	0.064*	
C14	0.7675 (5)	−0.0676 (4)	0.0483 (8)	0.0549 (17)	
H14	0.7645	−0.0667	−0.0635	0.066*	
C15	0.6836 (5)	−0.0422 (3)	0.1137 (7)	0.0467 (15)	
H15	0.6237	−0.0253	0.0462	0.056*	
C16	0.8780 (5)	0.1798 (3)	0.3887 (7)	0.0435 (14)	
C17	0.9578 (6)	0.2285 (4)	0.4050 (8)	0.0568 (17)	
H17	0.9518	0.2686	0.4630	0.068*	
C18	1.0448 (7)	0.2181 (5)	0.3372 (10)	0.072 (2)	
H18	1.0966	0.2518	0.3461	0.087*	
C19	1.0569 (6)	0.1583 (4)	0.2558 (9)	0.066 (2)	
H19	1.1176	0.1511	0.2119	0.080*	
C20	0.9785 (6)	0.1086 (4)	0.2390 (9)	0.0629 (19)	
H20	0.9865	0.0677	0.1848	0.075*	
C21	0.8876 (5)	0.1202 (4)	0.3038 (8)	0.0549 (17)	
H21	0.8336	0.0877	0.2898	0.066*	
H1	0.644 (6)	0.000 (8)	0.710 (16)	0.19 (6)*	

### Atomic displacement parameters (Å^2^)

**Table:**

	*U*^11^	*U*^22^	*U*^33^	*U*^12^	*U*^13^	*U*^23^
Se	0.0632 (5)	0.0332 (4)	0.0829 (6)	−0.0006 (3)	0.0221 (4)	−0.0055 (3)
S	0.0302 (7)	0.0479 (9)	0.0366 (8)	−0.0027 (6)	0.0020 (6)	0.0017 (6)
O1	0.038 (2)	0.041 (2)	0.046 (2)	−0.0011 (18)	0.0017 (19)	0.0029 (17)
O2	0.027 (2)	0.073 (3)	0.044 (2)	0.0022 (19)	−0.0048 (18)	0.001 (2)
C1	0.033 (3)	0.033 (3)	0.050 (4)	0.007 (2)	0.010 (3)	−0.002 (2)
C2	0.032 (3)	0.041 (3)	0.039 (3)	0.008 (2)	0.007 (2)	0.001 (2)
C3	0.054 (4)	0.050 (4)	0.044 (4)	0.006 (3)	0.010 (3)	0.005 (3)
C4	0.062 (4)	0.062 (5)	0.055 (4)	0.026 (4)	0.008 (3)	0.010 (3)
C5	0.069 (5)	0.070 (5)	0.071 (5)	0.018 (4)	0.001 (4)	0.018 (4)
C6	0.098 (7)	0.097 (8)	0.101 (7)	0.053 (6)	0.002 (6)	0.017 (5)
C7	0.031 (3)	0.049 (4)	0.044 (3)	−0.006 (3)	0.000 (3)	−0.004 (3)
C8	0.045 (4)	0.073 (5)	0.068 (5)	−0.014 (3)	−0.003 (3)	0.009 (4)
C9	0.073 (5)	0.065 (5)	0.055 (4)	−0.010 (4)	0.015 (4)	−0.015 (3)
C10	0.032 (3)	0.035 (3)	0.040 (3)	−0.004 (2)	0.003 (2)	0.001 (2)
C11	0.047 (3)	0.047 (4)	0.038 (3)	0.006 (3)	0.000 (3)	0.002 (3)
C12	0.045 (4)	0.063 (4)	0.051 (4)	0.019 (3)	−0.003 (3)	0.002 (3)
C13	0.048 (4)	0.055 (4)	0.058 (4)	0.018 (3)	0.012 (3)	0.002 (3)
C14	0.064 (4)	0.064 (5)	0.037 (3)	0.016 (3)	0.010 (3)	−0.001 (3)
C15	0.043 (3)	0.051 (4)	0.042 (3)	0.010 (3)	−0.002 (3)	−0.003 (3)
C16	0.047 (3)	0.032 (3)	0.050 (4)	−0.002 (3)	0.006 (3)	0.005 (3)
C17	0.063 (4)	0.044 (4)	0.060 (4)	−0.012 (3)	0.001 (4)	−0.002 (3)
C18	0.067 (5)	0.072 (5)	0.075 (5)	−0.024 (4)	0.004 (4)	0.020 (4)
C19	0.054 (4)	0.081 (6)	0.067 (5)	−0.003 (4)	0.017 (4)	0.011 (4)
C20	0.061 (4)	0.064 (5)	0.064 (5)	0.008 (4)	0.014 (4)	0.004 (4)
C21	0.053 (4)	0.048 (4)	0.065 (4)	−0.008 (3)	0.015 (3)	−0.005 (3)

### Geometric parameters (Å, º)

**Table:**

Se—C16	1.930 (6)	C8—H8C	0.9600
Se—C1	1.945 (6)	C9—H9A	0.9600
S—O2	1.499 (4)	C9—H9B	0.9600
S—C10	1.793 (6)	C9—H9C	0.9600
S—C2	1.844 (6)	C10—C15	1.386 (8)
O1—C7	1.422 (7)	C10—C11	1.392 (8)
O1—H1	0.85 (2)	C11—C12	1.371 (9)
C1—C2	1.340 (8)	C11—H11	0.9300
C1—C7	1.534 (8)	C12—C13	1.385 (9)
C2—C3	1.523 (8)	C12—H12	0.9300
C3—C4	1.529 (9)	C13—C14	1.382 (9)
C3—H3A	0.9700	C13—H13	0.9300
C3—H3B	0.9700	C14—C15	1.385 (9)
C4—C5	1.510 (9)	C14—H14	0.9300
C4—H4A	0.9700	C15—H15	0.9300
C4—H4B	0.9700	C16—C21	1.378 (9)
C5—C6	1.514 (11)	C16—C17	1.385 (9)
C5—H5A	0.9700	C17—C18	1.357 (11)
C5—H5B	0.9700	C17—H17	0.9300
C6—H6A	0.9600	C18—C19	1.370 (12)
C6—H6B	0.9600	C18—H18	0.9300
C6—H6C	0.9600	C19—C20	1.387 (10)
C7—C9	1.530 (9)	C19—H19	0.9300
C7—C8	1.532 (8)	C20—C21	1.393 (9)
C8—H8A	0.9600	C20—H20	0.9300
C8—H8B	0.9600	C21—H21	0.9300
			
C16—Se—C1	100.2 (2)	H8A—C8—H8C	109.5
O2—S—C10	104.3 (2)	H8B—C8—H8C	109.5
O2—S—C2	104.7 (2)	C7—C9—H9A	109.5
C10—S—C2	95.0 (2)	C7—C9—H9B	109.5
C7—O1—H1	117 (10)	H9A—C9—H9B	109.5
C2—C1—C7	128.9 (5)	C7—C9—H9C	109.5
C2—C1—Se	117.6 (4)	H9A—C9—H9C	109.5
C7—C1—Se	113.5 (4)	H9B—C9—H9C	109.5
C1—C2—C3	125.9 (6)	C15—C10—C11	120.2 (5)
C1—C2—S	123.3 (4)	C15—C10—S	120.0 (4)
C3—C2—S	110.8 (4)	C11—C10—S	119.8 (4)
C2—C3—C4	111.9 (5)	C12—C11—C10	119.8 (6)
C2—C3—H3A	109.2	C12—C11—H11	120.1
C4—C3—H3A	109.2	C10—C11—H11	120.1
C2—C3—H3B	109.2	C11—C12—C13	120.6 (6)
C4—C3—H3B	109.2	C11—C12—H12	119.7
H3A—C3—H3B	107.9	C13—C12—H12	119.7
C5—C4—C3	112.4 (6)	C14—C13—C12	119.5 (6)
C5—C4—H4A	109.1	C14—C13—H13	120.2
C3—C4—H4A	109.1	C12—C13—H13	120.2
C5—C4—H4B	109.1	C13—C14—C15	120.6 (6)
C3—C4—H4B	109.1	C13—C14—H14	119.7
H4A—C4—H4B	107.9	C15—C14—H14	119.7
C4—C5—C6	113.6 (7)	C14—C15—C10	119.2 (5)
C4—C5—H5A	108.8	C14—C15—H15	120.4
C6—C5—H5A	108.8	C10—C15—H15	120.4
C4—C5—H5B	108.8	C21—C16—C17	119.5 (6)
C6—C5—H5B	108.8	C21—C16—Se	123.5 (5)
H5A—C5—H5B	107.7	C17—C16—Se	117.0 (5)
C5—C6—H6A	109.5	C18—C17—C16	120.7 (7)
C5—C6—H6B	109.5	C18—C17—H17	119.7
H6A—C6—H6B	109.5	C16—C17—H17	119.7
C5—C6—H6C	109.5	C17—C18—C19	120.7 (7)
H6A—C6—H6C	109.5	C17—C18—H18	119.6
H6B—C6—H6C	109.5	C19—C18—H18	119.6
O1—C7—C9	110.5 (5)	C18—C19—C20	119.7 (7)
O1—C7—C8	105.3 (5)	C18—C19—H19	120.2
C9—C7—C8	110.1 (6)	C20—C19—H19	120.2
O1—C7—C1	110.3 (4)	C19—C20—C21	119.7 (7)
C9—C7—C1	109.4 (5)	C19—C20—H20	120.2
C8—C7—C1	111.3 (5)	C21—C20—H20	120.2
C7—C8—H8A	109.5	C16—C21—C20	119.7 (6)
C7—C8—H8B	109.5	C16—C21—H21	120.1
H8A—C8—H8B	109.5	C20—C21—H21	120.1
C7—C8—H8C	109.5		
			
C16—Se—C1—C2	−84.4 (5)	C2—S—C10—C15	−86.9 (5)
C16—Se—C1—C7	95.7 (4)	O2—S—C10—C11	−160.4 (5)
C7—C1—C2—C3	174.0 (5)	C2—S—C10—C11	93.0 (5)
Se—C1—C2—C3	−5.9 (8)	C15—C10—C11—C12	2.1 (9)
C7—C1—C2—S	−4.4 (8)	S—C10—C11—C12	−177.8 (5)
Se—C1—C2—S	175.7 (3)	C10—C11—C12—C13	−0.3 (10)
O2—S—C2—C1	161.7 (5)	C11—C12—C13—C14	−0.9 (11)
C10—S—C2—C1	−92.1 (5)	C12—C13—C14—C15	0.1 (11)
O2—S—C2—C3	−16.9 (4)	C13—C14—C15—C10	1.7 (10)
C10—S—C2—C3	89.3 (4)	C11—C10—C15—C14	−2.8 (9)
C1—C2—C3—C4	−83.7 (8)	S—C10—C15—C14	177.1 (5)
S—C2—C3—C4	94.9 (6)	C1—Se—C16—C21	21.1 (6)
C2—C3—C4—C5	175.6 (6)	C1—Se—C16—C17	−158.8 (5)
C3—C4—C5—C6	−178.6 (8)	C21—C16—C17—C18	0.5 (10)
C2—C1—C7—O1	3.4 (8)	Se—C16—C17—C18	−179.6 (5)
Se—C1—C7—O1	−176.6 (4)	C16—C17—C18—C19	−2.1 (11)
C2—C1—C7—C9	−118.2 (6)	C17—C18—C19—C20	1.6 (12)
Se—C1—C7—C9	61.7 (6)	C18—C19—C20—C21	0.6 (11)
C2—C1—C7—C8	119.9 (7)	C17—C16—C21—C20	1.7 (10)
Se—C1—C7—C8	−60.1 (6)	Se—C16—C21—C20	−178.2 (5)
O2—S—C10—C15	19.7 (5)	C19—C20—C21—C16	−2.2 (10)

### Hydrogen-bond geometry (Å, º)

**Table:**

*D*—H···*A*	*D*—H	H···*A*	*D*···*A*	*D*—H···*A*
O1—H1···O2^i^	0.85 (9)	1.91 (8)	2.737 (6)	167 (10)
C11—H11···O1	0.93	2.50	3.180 (7)	130
C15—H15···O2^ii^	0.93	2.60	3.463 (7)	155

Symmetry codes: (i) −*x*+1, −*y*, −*z*+1; (ii) −*x*+1, −*y*, −*z*.
